# A Computational Pipeline for Activity Prediction Using Wearable Sensor Data

**DOI:** 10.21203/rs.3.rs-9359112/v1

**Published:** 2026-04-24

**Authors:** Joshua Chuah, Laia Vancells-Lopez, Amy K. Loya, Danushka Bandara, John F. Drazan

**Affiliations:** 1* Department of Electrical, Computer, and Biomedical Engineering, Union College, 807 Union St., Schenectady, 12308, NY, USA.; 2 Department of Biomedical Engineering, Purdue University, 610 Purdue Mall, West Lafayette, 47907, IN, USA.; 3 Department of Computer Science, Fairfield University, 1073 North Benson Road, Fairfield, 06824, CT, USA.; 3 Department of Biomedical Engineering, Fairfield University, 1073 North Benson Road, Fairfield, 06824, CT, USA.

**Keywords:** wearable sensor, biomechanics, machine learning

## Abstract

**Background:**

Wearable sensors enable collection of ground reaction force (GRF) data in real-world settings, but translating these data into meaningful activity classifications remains challenging, particularly for subject-specific monitoring.

**Results:**

We present a dataset of wearable GRF measurements from 14 subjects walking across 18 combinations of speed and incline, along with a machine learning pipeline for step-level classification of loading behaviors. Continuous GRF signals were segmented into individual gait cycles and transformed into features using TSFRESH, followed by feature selection and Random Forest classification. Subject-specific models achieved a mean Top-1 accuracy of 0.664 (SD = 0.053), exceeding chance performance (0.056), with Top-2 and Top-3 accuracies of 0.836 and 0.904, respectively. Accuracy remained similar for incline-only classification (0.688 ± 0.030) but increased for speed-only classification (0.903 ± 0.097).

**Conclusions:**

These results demonstrate that step-level GRF data can support accurate classification of locomotion-related loading conditions and enable the development of subject-specific models for monitoring individual activity. The dataset and pipeline provide a foundation for future work in wearable biomechanics and personalized analysis of musculoskeletal loading.

## 1 Introduction

Understanding musculoskeletal tissue loading during locomotion is critical for injury prevention and rehabilitation monitoring. Improper or excessive loading can lead to pain, swelling, and impaired function of certain muscles and tendons [1]. Specifically, the Achilles tendon can be particularly sensitive to changes in loading conditions on the foot associated with walking speed or incline, and injuries can be caused by behaviors including training error, activity intensity increases, or inappropriate footwear [2, 3]. As such, it is important to relate real-world activities to externally measured forces on the foot, which are associated with musculoskeletal loading. This is essential for monitoring loading exposure and guiding clinical interventions. If these forces can be reliably associated with specific activities, clinicians may better identify harmful movement patterns and tailor rehabilitation strategies.

To enable this, researchers must create models that link measured ground reaction forces (GRFs) to the underlying activity being performed [4, 5]. To create these models, consequently, there must be frameworks to collect and analyze large sets of physiological and mechanical data from activities of daily living. However, traditional methods of data collection typically use laboratory tools that restrict the ability to form in-depth mechanistic understandings of musculoskeletal pathology [6, 7]. While wearable sensors mitigate some of these problems by being able to collect data outside of the laboratory, allowing the patient to mimic their normal, everyday behaviors, overcoming limitations of wearable sensors unable to estimate internal tissue-level loading remains a challenge [8]. Moreover, it is often difficult for researchers to automatically identify individual steps while patients are wearing these sensors, leading to a time-intensive manual parsing process to preprocess the data. To bridge this gap, researchers utilize mathematical models such as inverse kinematics and dynamics, exemplified by calculating plantar flexion moments using approximated moment arms and exerted loads[9]. Although these models are valuable, data collected from wearable sensors is often complex, encompassing noisy, large and time dependent force measurements. For this reason, the development of computational methods to accurately and automatically analyze large data sets of GRF is imperative.

Machine learning has been used to automate the analysis of certain wearable sensor datasets, making it an attractive tool for high-throughput GRF data processing and analysis. Matijevich et. al used wearable sensor feature to create a regression model to estimate tibial force and bone damage during running, with low error rates [10]. Furthermore, machine learning shows promise in addressing the typical problems associated with wearable sensor analysis by automating the processing of wearable sensor datasets [11]. As such, recent research has shown that it is possible to develop models that can predict falls, internal forces, and risk of injury from wearable sensor data [12–14]. The combination of wearable sensors and machine learning allows for continuous monitoring of musculoskeletal function outside clinical settings, providing insights into daily activities, exercise routines, and rehabilitation progress. This continuous monitoring can facilitate early detection of musculoskeletal issues, track recovery trajectories, and optimize interventions to improve outcomes [15]. As such, the need for a workflow that allows researchers to create machine learning models that can accurately detect certain loading behaviors is imperative.

Despite these advances, most machine learning approaches in biomechanics are developed as population-level models. However, biomechanical data acquired from wearable sensors can vary substantially between individuals due to heterogeneity in gait, anatomy and sensor placement. As a result, population-level models may fail to capture subject-specific trends. The development of personalized, subject-specific models using a small baseline dataset may enable more accurate monitoring of loading conditions.

In this study, we present a computational pipeline for processing wearable sensor GRF data and predicting locomotion conditions, specifically walking speed and incline. The proposed approach uses subject-specific baseline data to train individualized models capable of classifying activity from GRF waveforms. The main contributions of this work are: (1) the introduction of a large, labeled wearable sensor dataset for locomotion analysis, and (2) the development of a subject-specific machine learning pipeline for activity classification from GRF data.

## 2 Methods

### 2.1 Pipeline

**Fig. 1:**
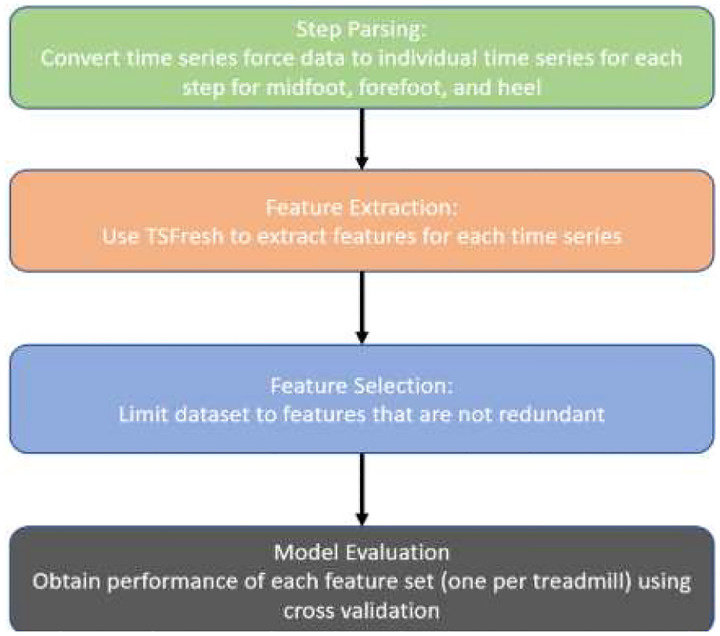
General workflow for the proposed pipeline.

**Fig. 2:**
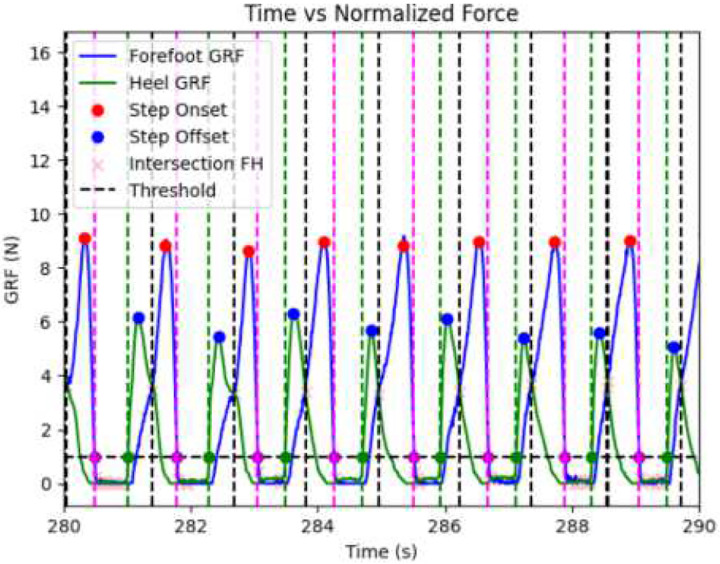
Parsing of the longitudinal GRF data into individual steps. The blue line tracks the force exhibited by the forefoot, and the green line shows that exhibited by the heel. The peak of the heel force is used as the step offset, while the peak of the forefoot force is used as the step onset, e.g. where the foot leaves and makes contact with the ground, respectively. Steps are recorded as beginning where the heel data increases across the predetermined threshold, and ending where the forefoot data decreases back across this threshold.

The data analysis pipeline can be broken down into four main parts, symbolized in Figure 1: 1) a step parsing algorithm, 2) feature extraction, 3) feature selection, and 4) model evaluation. The step parsing algorithm first identifies and labels individual steps using a novel algorithm that combined peaks and intersection detection. Gait cycles were segmented by detecting biomechanical phase transitions from normalized GRF signals of the forefoot (S_FF_), heel (S_H_), and total foot (S_T_). Phase transitions were identified using intersection analysis, defined as time points where the sign of the pointwise difference between two GRF signals reversed between consecutive samples. Three physiologically relevant crossings were retained: (1) Initial heel contact, where S_H_ = S_T_; (2) Midstance, where S_FF_ = S_H_; and (3) Toe-off, where S_FF_ = S_T_. Intersections were refined to those in which one signal approached zero amplitude at the crossing, ensuring precise stance–swing demarcation. Discrete steps were further labeled via peak detection on S_FF_ and S_H_ trajectories. Forefoot peaks were defined as local maxima in S_FF_ exceeding 7 N and separated by ≥ 80 ms; heel peaks as local maxima in SH exceeding 3.5 N with the same interval. Thresholds were empirically optimized across all subjects to maximize sensitivity to true gait events while minimizing noise-related false positives. Heel strikes and toe-offs, demarcating individual step boundaries, were identified using threshold-crossing logic anchored to previously detected GRF peaks to ensure correct temporal sequencing. A heel strike was defined as the first S_H_ crossing above 1 N with a positive slope prior to the identified heel peak, whereas a toe-off was defined as the first S_FF_ crossing below 1 N with a negative slope subsequent to the detected forefoot peak. Each gait cycle was defined as the interval between heel strike and the following toe-off and assigned a unique step identifier. All detected gait events were validated by visual inspection of synchronized GRF trajectories across the full recording duration to confirm correct delineation of steps, including peak detections and intersection points, and to exclude false-positive detections (Fig. 2).

This process identified three key intersections: 1) The initial heel contact phase, 2) the midstance phase, and 3) the toe-off phase. To ensure precision: minimum peak and height distances were established to eliminate redundancy, and values below 1.5 N/Body weight were disregarded to filter out noise without compromising data integrity. This approach captures key temporal characteristics of gain, such as step duration, step length, and step symmetry, enabling a detailed accounting of gait quality as preserved in the resulting data frame. Moreover, an additional layer of data refinement has been implemented to enhance the precision and specificity of our gait analyses. After the GRF data was preprocessed; the heel, forefoot, and midfoot data were combined to calculate the approximate force applied to the Achilles tendon at each time point. These distinctions in phase can be seen in Figure 2. Preprocessed data files were then saved and prepared for the machine learning model.

After segmenting individual steps into time-series measurements, feature extraction was performed using the TSFRESH Python package [16]. Each step initially consisted of three ground reaction force (GRF) signals (forefoot, midfoot, and heel) measured across time. To enable the use of conventional machine learning models and to capture descriptive characteristics of the temporal signals, each time series was transformed into a set of summary features describing its statistical, temporal, and frequency-domain properties. This process expanded each step from three raw signals into a feature vector containing over 3000 candidate features. Features containing non-continuous data, missing values, or NaN elements were removed prior to further analysis.

Due to the fact TSFRESH generates a large number of features, feature selection is necessary to prevent overfitting of the resulting classification model. The goal of feature selection in this workflow was 1) to limit the amount of redundant features, therefore reducing the risk of overfitting, and 2) to ensure that the features kept were most important in predicting activity from the extracted, time-series features [17]. The former was performed by the Smart Correlated feature selection module from the feature engine python package[18, 19]. This feature selection method finds highly correlated features in the dataset, and determines which ones to keep based on the performance of a single feature machine learning model trained by only that feature’s data. Low performing features are removed from further consideration to remove highly correlated, i.e., redundant variables. The latter was performed by identifying the top *K* features by computed f-score, i.e. features whose measurements showed differences based on predicted activity [20]. The value of *K* was selected by computing the 5-fold cross-validation accuracy on the training data for models trained with features at different levels of *K*. The chosen value of *K* was the smallest *K* (i.e., the least complex model) whose cross-validation accuracy was within one standard error of the highest cross-validation accuracy observed [21]. This enabled feature selection which took measures to prevent overfitting and find accurate selections of variables while not requiring the same features to be selected each time, allowing for subject-specific models.

Finally, a machine learning model is trained on the remaining features, with each class label corresponding to a loading behavior (e.g., combination of speed and incline). In this study, 5-fold cross-validation was selected to balance computational efficiency and robustness of performance estimation given that there were hundreds of samples per subject in this dataset. It is important to note that 5-fold cross validation was used in lieu of a more conservative validation procedure such as leave-one-out cross-validation to decrease the computational complexity of the experiment. However, if the classifiers can be trained efficiently and there are few samples (steps) in the dataset, higher amounts of folds can be considered.

### 2.2 Case Study

This study demonstrates the use of the proposed pipeline on a new dataset of 14 healthy adults (6 male, 8 females; 31+− 4 years; BMI 27.1 +− 6 kg/m2) who performed treadmill walking at a range of inclines and speed conditions In total, longitudinal step data at 6 inclines (0°, 5°, 10°, 15°, 20°, 25°) and 3 speeds (0.8 m/s, 1.2 m/s, 1.6 m/s) was measured for each participant, yielding 18 distinct combinations of loading conditions. Participants first underwent a standardized warm-up and cooldown, followed by treadmill walking for 30 seconds at each condition: 20 seconds of data collection following a 10-second adjustment period. Ground reaction forces were measured using the Loadsol wearable sensor at 100 Hz and were normalized by body weight, resulting in 3 variables of data (midfoot, heel, and forefoot) for all measured time points. Using each step as a sample and the GRF data columns as features, this workflow was followed for each of the 14 participants, e.g. 14 separate models were made to show that this workflow was subject-specific. Informed consent was obtained from all subjects involved in the study.

After loading the data files, warm-up and cool-down data were filtered out as they were not relevant to the classification task. The time series data was then grouped into ‘Forefoot’, ‘Midfoot’, and ‘Heel’ groups which correspond to loading in those regions of the foot.

The analysis pipeline was applied spearately to each participant to evaluate subject-specific model performance, resulting in 14 independent classification models. Individual steps were treated as samples, with GRF time-series measurements serving as features. There were 18 total loading conditions among these data points, including 3 walking speeds and 6 walking angles. A label encoder was used to convert categorical condition labels into numeric values. Time-series sequences corresponding to each step were zero-padded to match the length of the longest sequence. Data were then normalized to the range [0,1]to improve model training. For each participant, the dataset was partitioned such that 80% of the steps were randomly selected for feature selection (via cross-validation) and model training, while the remaining 20% were reserved for testing. From the GRF data, features were extracted using TSFresh, and features were selected using a combination of correlation-based and importance-based feature selection as detailed above, only using the training data. Random Forest classifiers were trained for each subject to capture potential nonlinear relationships among features, particularly given that the selected feature sets differed between individuals. Features were selected to optimize performance of each model on the 18-class problem. To evaluate the relative contributions of speed and incline angle to model performance, models with the same feature sets, input data and algorithm were retrained and evaluated for prediction of speed only (3 classes) and incline angle only (6 classes). To further assess prediction uncertainty and potential overlap among labels of the 18-class problem, top-2 and top-3 accuracy were also computed, where Top-k accuracy represents whether the correct class appears within the model’s k highest predicted probabilities.

## 3 Results

**Fig. 3:**
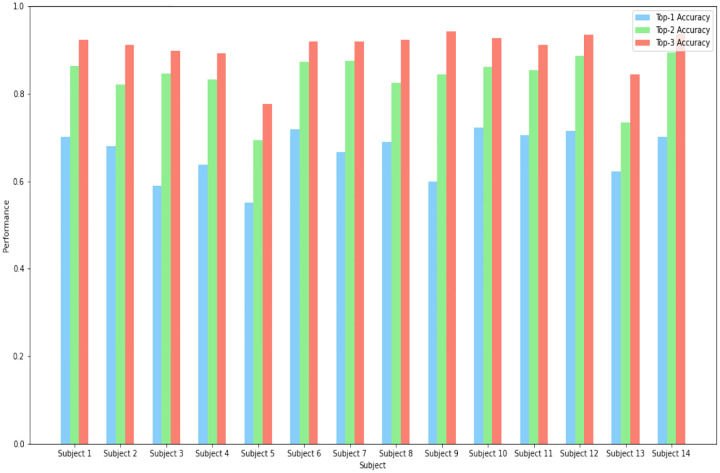
Comparison of the Top-1, Top-2 and Top-3 Accuracy for each model

For each of the 14 subjects, models were developed to predict loading behavior using GRF data from wearable sensors. There were 18 combinations of speed and incline for each model to predict. Figure 3 summarizes the Top-1, Top-2 and Top-3 accuracy of these models. Table 1 shows a complete breakdown of performance metrics for each of the subject-specific models. On average, the models were able to correctly identify the loading behavior the majority of the time with an average accuracy of 0.664 (median = 0.684) across 14 models, showing a predictive relationship between the features extracted and selected and predicted labels. Models across all subjects performed similarly, with a standard deviation in performance of 0.053. Furthermore, for an 18-class problem the expected accuracy of random selection is 1/18 = 0.0556, and each of these models substantially outperform this random accuracy. The Top-2 accuracy was vastly improved from the Top-1 accuracy, yielding an average performance of 0.836 (median = 0.850). Furthermore, the Top-3 performance yielded an average accuracy of 0.904 (median = 0.919), meaning that the correct label was among the 3 highest predicted probabilities from the model ¿90% of the time. Dispersion across subject-specific models was similar for the Top-2 model (0.054) and decreased for the Top-3 (0.043) model, indicating higher precision among the models.

**Fig. 4:**
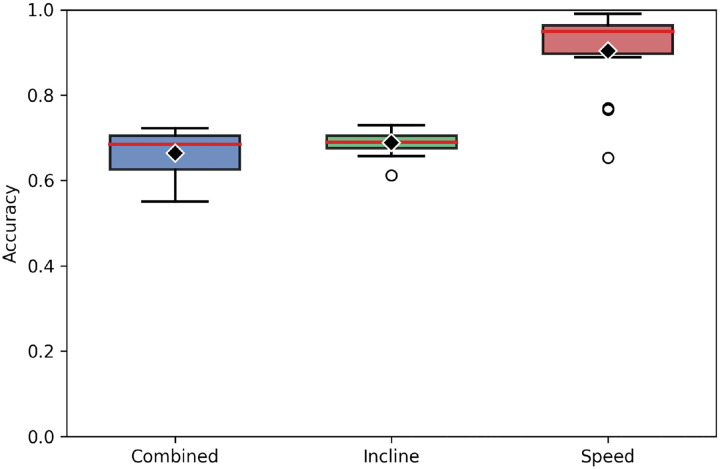
Comparison of model performances across 14-subjects when predicting all 18 combinations of loading behaviors (blue), walking incline angle only (green), and walking speed only (red)

Additionally, each subject’s model was retrained and retested to determine whether incline angle and walking speed could be predicted independently rather than as a combined 18-class problem. When predicting incline angle alone, model performance was similar to that observed for the combined classification task, with an average accuracy of 0.688 ± 0.030 (mean ± SD; median = 0.689) across the 14 subject-specific models. When predicting walking speed alone, however, model performance increased substantially. The average accuracy across all models was 0.903 ± 0.097, with a median accuracy of 0.950, indicating that speed classification was generally more accurate than incline prediction. Performance variability across subjects was also greater for the speed prediction task, as reflected by the larger standard deviation. These changes in summary performance and variability are visualized in Figure 4. In general, models trained with larger numbers of samples tended to exhibit higher prediction accuracy, although this trend was not consistent across all subjects.

Precision and recall of each model were also evaluated to determine if accuracy results were skewed by a particular label being overrepresented and favored by the classification model. Weighted-averaged precision across each subject-specific model was 0.672, while the weighted-average recall was 0.664. Due to the fact that these are similar to the accuracy, suggesting that model performance was balanced in terms of true positive and true negative rate.

## 4 Discussion

This study presents a wearable sensor dataset and a computational pipeline for classifying loading behaviors from ground reaction force (GRF) data. The results demonstrate that step-level GRF signals contain sufficient information to support activity classification and that subject-specific machine learning models can be trained to identify loading conditions with performance above chance. Collectively, these findings support the feasibility of using wearable sensor data for individualized monitoring of locomotor loading outside of laboratory environments.

A primary contribution of this work is the introduction of a labeled dataset of wearable GRF measurements collected across a range of walking speeds and inclines. The dataset captures step-level variations in forefoot, midfoot, and heel loading, enabling the development and evaluation of classification models based on physiologically meaningful signals. By structuring the data at the level of individual gait cycles, this work provides a resource that can be used to investigate relationships between locomotion conditions and loading patterns.

The step parsing approach used in this study further supports the relationship between GRF signals and locomotion conditions. By segmenting continuous data into discrete gait cycles using a combination of intersection detection and peak-based methods, the pipeline is able to accurately isolate steps. While step-parsing was validated through visual inspection, the ability of the models to clearly identify steps taken during specific activities also indirectly reflects the validation of these steps.

Across subjects, classification models achieved moderate to high Top-1 accuracy for the 18-class loading condition task, with substantially higher Top-2 and Top-3 accuracies. The improvement in Top-k performance suggests that when misclassifications occur, the correct label is often ranked among the most probable predictions. This pattern is consistent with the presence of overlapping or similar GRF signatures between certain loading conditions, particularly those involving adjacent incline angles. This interpretation is further supported by the observation that predicting incline angle alone did not substantially improve accuracy relative to the combined classification task, despite the reduced number of classes. In contrast, prediction of walking speed alone resulted in higher accuracy, even after accounting for the smaller number of labels. Together, these findings suggest that GRF-derived features more distinctly capture variations in walking speed than incline, while incline-related differences may produce more subtle or overlapping signal characteristics that increase classification difficulty.

Importantly, this study demonstrates the feasibility of training subject-specific models using wearable GRF data. By developing independent models for each participant, the analysis accounts for inter-individual variability in gait patterns, anatomy, and sensor placement. The relatively consistent performance (small standard deviation) observed across subjects indicates that the proposed pipeline can be applied at the individual level without requiring population-level generalization. This subject-specific framework is particularly relevant for applications in rehabilitation and longitudinal monitoring, where the goal is to track changes within an individual over time rather than to generalize across populations. The ability to construct personalized models from baseline data supports the potential use of wearable sensors for continuous, real-world monitoring of locomotor behavior.

Several considerations should be noted when interpreting these results. First, while individual steps were treated as samples for model training and evaluation, steps collected within a continuous walking trial are temporally correlated and therefore not strictly independent. As a result, step-level classification may lead to optimistic estimates of model performance, particularly when training and test samples are drawn from the same recording sessions [22]. Feature extraction transforms each gait cycle into a set of summary descriptors, which reduces but does not eliminate temporal dependence between samples [16]. This approach is consistent with prior work in gait analysis, where individual steps are commonly treated as the unit of analysis despite underlying sequential structure [23, 24]. In this study, models were developed separately for each subject to reflect the intended use case of subject-specific monitoring, in which a model is trained on baseline data from an individual and applied to similar future observations from that same individual. Under this framework, the reported performance reflects accuracy within a subject under comparable conditions, rather than fully independent generalization across time or experimental sessions. Additionally, the dataset consists of controlled treadmill walking conditions in healthy adults; therefore, further work is needed to evaluate how these models generalize to more variable or rapidly changing real-world activities.

## 5 Conclusion

In summary, this study demonstrates that wearable GRF data can be used to train subject-specific machine learning models capable of classifying locomotion-related loading behaviors at the level of individual steps. The goal of this work was not to estimate fully independent generalization across subjects or sessions, but rather to evaluate the feasibility of constructing accurate subject-specific classifiers from wearable GRF data under controlled conditions. The combination of a structured dataset and a reproducible analysis pipeline provides a foundation for future work in wearable biomechanics and personalized monitoring of musculoskeletal loading. Future work may extend this approach by validating this pipeline with additional datasets and exploring methods for improving discrimination between similar loading conditions, particularly for walking incline classification.

## Figures and Tables

**Table 1:** Subject-specific model performance. Top-*K* refers to the accuracy of the model when the model computes a prediction probability of the correct class within the Top-*K* computed predicted probabilities.

Subject No.	Sample Size	Top-1	Top-2	Top-3	Precision	Recall
1	585	0.701	0.863	0.923	0.711	0.701
2	666	0.679	0.821	0.910	0.677	0.679
3	389	0.590	0.846	0.897	0.585	0.590
4	415	0.639	0.831	0.891	0.653	0.639
5	241	0.551	0.694	0.776	0.575	0.551
6	549	0.718	0.873	0.918	0.710	0.718
7	553	0.667	0.874	0.919	0.649	0.667
8	515	0.689	0.826	0.922	0.733	0.689
9	609	0.598	0.844	0.943	0.589	0.598
10	536	0.722	0.861	0.926	0.748	0.722
11	509	0.706	0.853	0.912	0.738	0.706
12	523	0.714	0.886	0.933	0.742	0.714
13	222	0.622	0.733	0.844	0.579	0.622
14	234	0.702	0.894	0.936	0.719	0.702
Average	468	0.664	0.836	0.904	0.672	0.664

## Data Availability

Data is available by request from John F. Drazan. All code used to generate the results in this study can be accessed at: https://github.com/ChuahResearchGroup/AchillesTendon

## References

[1] Carlson J.M.: Functional limitations from pain caused by repetitive loading on the skin: a review and discussion for practitioners, with new data for limiting friction loads. JPO: Journal of Prosthetics and Orthotics 18(4), 93–103 (2006)

[2] Maganaris C.N., Narici M.V., Maffulli N.: Biomechanics of the achilles tendon. Disability and rehabilitation 30(20–22), 1542–1547 (2008) 18720120 10.1080/09638280701785494

[3] Paavola M., Kannus P., Järvinen T.A., Khan K., Józsa L., Järvinen M.: Achilles tendinopathy. JBJS 84(11), 2062–2076 (2002) 10.2106/00004623-200211000-00024 12429771

[4] Fluit R., Andersen M.S., Kolk S., Verdonschot N., Koopman H.F.: Prediction of ground reaction forces and moments during various activities of daily living. Journal of biomechanics 47(10), 2321–2329 (2014) 24835471 10.1016/j.jbiomech.2014.04.030

[5] Fahmie T.A., Rodriguez N.M., Luczynski K.C., Rahaman J.A., Charles B.M., Zangrillo A.N.: Toward an explicit technology of ecological validity. Journal of Applied Behavior Analysis 56(2), 302–322 (2023) 36717983 10.1002/jaba.972

[6] Drazan J.F., Phillips W.T., Seethapathi N., Hullfish T.J., Baxter J.R.: Moving outside the lab: Markerless motion capture accurately quantifies sagittal plane kinematics during the vertical jump. Journal of Biomechanics 125, 110547 (2021) 34175570 10.1016/j.jbiomech.2021.110547 PMC8640714

[7] Ferber R., Osis S.T., Hicks J.L., Delp S.L.: Gait biomechanics in the era of data science. Journal of biomechanics 49(16), 3759–3761 (2016) 27814971 10.1016/j.jbiomech.2016.10.033 PMC5407492

[8] Kobsar D., Charlton J.M., Tse C.T., Esculier J.-F., Graffos A., Krowchuk N.M., Thatcher D., Hunt M.A.: Validity and reliability of wearable inertial sensors in healthy adult walking: A systematic review and meta-analysis. Journal of neuroengineering and rehabilitation 17(1), 62 (2020) 32393301 10.1186/s12984-020-00685-3 PMC7216606

[9] Hullfish T.J., Baxter J.R.: A simple instrumented insole algorithm to estimate plantar flexion moments. Gait & posture 79, 92–95 (2020) 32388057 10.1016/j.gaitpost.2020.04.016

[10] Matijevich E.S., Scott L.R., Volgyesi P., Derry K.H., Zelik K.E.: Combining wearable sensor signals, machine learning and biomechanics to estimate tibial bone force and damage during running. Human movement science 74, 102690 (2020) 33132194 10.1016/j.humov.2020.102690 PMC9827619

[11] Halilaj E., Rajagopal A., Fiterau M., Hicks J.L., Hastie T.J., Delp S.L.: Machine learning in human movement biomechanics: Best practices, common pitfalls, and new opportunities. Journal of biomechanics 81, 1–11 (2018) 30279002 10.1016/j.jbiomech.2018.09.009 PMC6879187

[12] Elstub L., Nurse C., Grohowski L., Volgyesi P., Wolf D., Zelik K.: Tibial bone forces can be monitored using shoe-worn wearable sensors during running. Journal of sports sciences 40(15), 1741–1749 (2022) 35938189 10.1080/02640414.2022.2107816 PMC9938946

[13] Özdemir A.T., Barshan B.: Detecting falls with wearable sensors using machine learning techniques. Sensors 14(6), 10691–10708 (2014) 24945676 10.3390/s140610691 PMC4118339

[14] Johnson W.R., Mian A., Robinson M.A., Verheul J., Lloyd D.G., Alderson J.A.: Multidimensional ground reaction forces and moments from wearable sensor accelerations via deep learning. IEEE Transactions on Biomedical Engineering 68(1), 289–297 (2020) 32746046 10.1109/TBME.2020.3006158

[15] Muzaffar S., Elfadel I.M.: Self-synchronized, continuous body weight monitoring using flexible force sensors and ground reaction force signal processing. IEEE Sensors Journal 20(18), 10886–10897 (2020)

[16] Christ M., Braun N., Neuffer J., Kempa-Liehr A.W.: Time series feature extraction on basis of scalable hypothesis tests (tsfresh–a python package). Neurocomputing 307, 72–77 (2018)

[17] Li J., Cheng K., Wang S., Morstatter F., Trevino R.P., Tang J., Liu H.: Feature selection: A data perspective. ACM computing surveys (CSUR) 50(6), 1–45 (2017)

[18] Galli S.: Feature-engine: A python package for feature engineering for machine learning. Journal of Open Source Software 6(65), 3642 (2021)

[19] Hall M.A.: Correlation-based feature selection for machine learning. PhD thesis, The University of Waikato (1999)

[20] Saeed M.H., Hama J.I.: Cardiac disease prediction using ai algorithms with selectkbest. Medical & Biological Engineering & Computing 61(12), 3397–3408 (2023) 37679578 10.1007/s11517-023-02918-8

[21] Hastie T., Tibshirani R., Friedman J.H., Friedman J.H.: The Elements of Statistical Learning: Data Mining, Inference, and Prediction vol. 2. Springer, ??? (2009)

[22] Hassani H., Royer-Carenzi M., Mashhad L.M., Yarmohammadi M., Yeganegi M.R.: Exploring the depths of the autocorrelation function: its departure from normality. Information 15(8), 449 (2024)

[23] Khera P., Kumar N.: Role of machine learning in gait analysis: a review. Journal of Medical Engineering & Technology 44(8), 441–467 (2020) 33078988 10.1080/03091902.2020.1822940

[24] Ordóñez F.J., Roggen D.: Deep convolutional and lstm recurrent neural networks for multimodal wearable activity recognition. Sensors 16(1), 115 (2016) 26797612 10.3390/s16010115 PMC4732148

